# First Complete Genome Sequence of *Papaya ringspot* *virus*-W Isolated from a Gourd in the United States

**DOI:** 10.1128/genomeA.01434-16

**Published:** 2017-01-12

**Authors:** Akhtar Ali

**Affiliations:** Department of Biological Science, The University of Tulsa, Tulsa, Oklahoma, USA

## Abstract

In the United States, the *Papaya ringspot virus* was first reported from papaya in Florida in 1949. Here, we determined the first complete genome sequence (10,302 nucleotides) of a *Papaya ringspot virus*-W isolate, which was collected from a commercial field of gourd in Tulsa, OK.

## GENOME ANNOUNCEMENT

Cucurbits are grown worldwide and are economically important because of their contribution to our diet. In the United States, the cultivation of cucurbits is mostly concentrated in the southern states (1), where a number of viruses have been reported from cucurbit crops (2–7). Among the viruses, *Papaya ringspot virus*-W (PRSV-W; genus *Potyvirus*, family *Potyviridae*) is one of the most destructive potyviruses and is a limiting factor for cucurbit production worldwide (8).

PRSV has two different strains (P and W) based on their infectivity of different hosts. PRSV-P infects papaya, while PRSV-W infects cucurbits. In the United States, the occurrence of both strains has been reported from papaya and cucurbits (9, 10). The complete genome of a PRSV-P strain from Hawaii has been reported. However, no information exists on the complete genome sequence of PRSV-W from cucurbits in the United States.

In Oklahoma, the incidence of PRSV-W was reported to be more than 50% in cucurbit fields (6), while it was 24% in other southern states (7). Although it is an important virus, to our knowledge, no complete genome of PRSV-W from any cucurbit crops has ever been sequenced in the United States. Here, in this work, the first complete genome sequence was determined of a PRSV-W isolate collected in a commercial gourd field in Tulsa, OK, USA.

During our previous studies on cucurbits viruses (6, 7), a PRSV-W-positive sample from gourd collected from a grower field in Tulsa County, OK, known as TUL15, showed severe mosaic and mottling and was selected for this study. The TUL15 isolate was maintained in a growth chamber by mechanically inoculating pumpkin seedlings using extracted sap from infected gourd leaves.

Two to three weeks postinoculation, total RNA was extracted according to the Tri-Reagent procedure (6) from systemically infected leaves of pumpkin seedlings infected with the PRSV-W TUL15 isolate. At least nine pairs of overlapping primers were designed from the complete genome sequences of PRSV isolates available from GenBank (http://www.ncbi.nlm.nih.gov). Nine genome fragments were amplified by the reverse transcription-PCR (RT-PCR) with respective primer pairs using total RNA as a template, as described previously (6). PCR products were confirmed on 1% agarose gels and cloned into pGEM-T Easy vector. Three to five recombinant clones were sequenced in both directions using Applied Biosystems 3130 at The University of Tulsa, OK.

All the nucleotide sequences were aligned using EditSeq and MEGAlign within DNAStar Lasergene (Madison, WI), and consensus sequences were obtained by the Clustal X program (11). The complete genome of the PRSV-W TUL15 isolate was generated by joining the overlapping sequences and was 10,302 nucleotides long, excluding the 3′-terminal poly(A) tail.

A nucleotide BLAST search using the complete genome of PRSV-W-TUL15 showed 83 to 92% nucleotide and 90 to 95% amino acid sequence similarities with the published PRSV isolates. Maximum nucleotide (92%) and amino acid (95%) sequence similarities were observed with a PRSV-W French E2 isolate (GenBank accession no. KC345609) (12).

### Accession number(s).

The complete genome sequence of the PRSV-W TUL15 isolate was deposited in GenBank under the accession no. KY039583.
